# Challenges for Elder Abuse Prevention Law

**DOI:** 10.1093/geroni/igaa057.2377

**Published:** 2020-12-16

**Authors:** Noriko Tsukada, Asako Katsumata

**Affiliations:** 1 Nihon University, Tokyo, Tokyo, Japan; 2 Rehabilitation Institution, Chiba, Tokyo, Japan

## Abstract

This paper compares components of four abuse prevention laws in Japan, including elder abuse, child abuse and domestic abuse, and abuse for people with disabilities and delineates major strengths and weaknesses of the Elder Abuse Prevention Law in comparison to the other three. Based on this analysis, this paper recommends improvements　in the elder abuse prevention law given the success of the related abuse laws. Despite the requirement of re-evaluation every 3 years, no amendments have been made to the elder abuse prevention law, while amendments have been made to the child abuse and domestic abuse prevention laws, based on outcome data and implementation experience. Identified needed revisions include provisions of protection orders and temporary shelters to protect elder victims from abusers at the time abuse is reported.

